# Genotypic Antimicrobial Resistance Profiles of Diarrheagenic *Escherichia coli* and Nontyphoidal *Salmonella* Strains Isolated from Children with Diarrhea and Their Exposure Environments in Ethiopia

**DOI:** 10.2147/IDR.S480395

**Published:** 2024-11-09

**Authors:** Dinaol Belina, Tesfaye Gobena, Ameha Kebede, Meseret Chimdessa, Tine Hald

**Affiliations:** 1 College of Veterinary Medicine, Haramaya University, Dire Dawa, Ethiopia; 2 School of Biological Sciences and Biotechnology, Haramaya University, Dire Dawa, Ethiopia; 3 College of Health and Medical Sciences, Haramaya University, Harar, Ethiopia; 4 National Food Institute, Technical University of Denmark, Lyngby, Denmark

**Keywords:** antimicrobial resistance, Ethiopia, foodborne pathogens, whole genome sequence

## Abstract

**Background:**

Antimicrobial resistance (AMR) poses a significant global threat, particularly in low- and middle-income countries, such as Ethiopia, where surveillance is limited. This study aimed to predict and characterize the AMR profiles of diarrheagenic *Escherichia coli* (DEC) and nontyphoidal *Salmonella* (NTS) strains isolated from human, animal, food, and environmental samples using whole genome sequencing.

**Methods:**

A total of 57 NTS and 50 DEC isolates were sequenced on an Illumina NextSeq 550. The *ResFinder* and *PointFinder* tools were employed to identify antimicrobial resistance genes (ARGs) and point mutations. *Salmonella* serotypes were determined using *SeqSero*.

**Results:**

The analysis identified at least one ARG in every NTS sample and 78% of the DEC isolates, with 22 distinct ARGs in the NTS samples and 40 in the DEC samples. The most prevalent ARGs were *aac*(6′)-Iaa and *aph*(3′)-Ib, which predict aminoglycoside resistance in 100% of NTS and 54% of DEC isolates, respectively. Other commonly identified ARGs include *sul*2, *aph*(6)-Id, *bla_TEM-1B_*, and *tet*(A), which confer resistance to folate inhibitors, aminoglycosides, β-lactams, and tetracycline. Some ARGs predicted phenotypic multidrug resistance in both DEC and NTS isolates. All identified β-lactam ARGs, except for *bla_TEM_*_−1D_, conferred resistance to more than three antibiotics. Interestingly, *bla_CTX-_*_M-15_ was found to confer resistance to nine antibiotics, including third-generation cephalosporins, in 18% of DEC and 3.5% of NTS isolates. DEC isolates from children exhibited the highest ARG diversity. Notably, genes such as *aph*(3″)-Ib, *aph*(6)-Id, *sul*2, and *tet*(A) were detected across all sample types, including water sources, although some ARGs were exclusive to specific sample types. Point mutations mediating AMR were detected in several genes, with mutations associated with nucleotide substitution being the most frequent.

**Conclusion:**

This genotypic AMR profiling revealed the presence of widespread drug-resistant NTS and DEC strains in Ethiopia. Robust and sustained AMR surveillance is essential for monitoring the emergence and spread of these resistant pathogens.

## Video abstract

**Figure:**
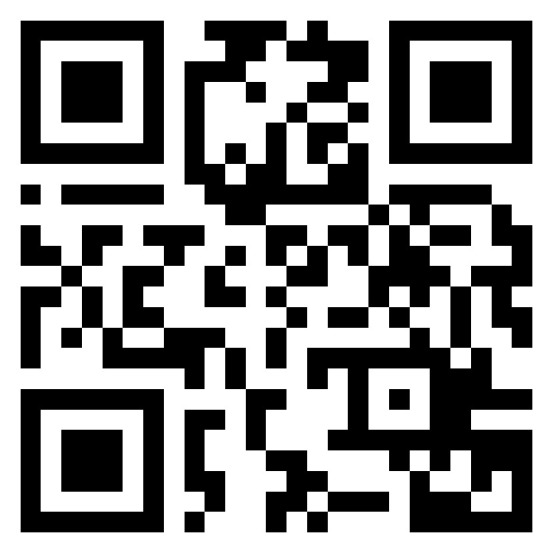

Point your SmartPhone at the code above. If you have a QR code reader the video abstract will appear. Or use: https://youtu.be/kJr6-N7yi0Y

## Introduction

Antimicrobial resistance (AMR) is a pressing global public health concern that affects treatment outcomes across all regions and income levels. It is estimated that AMR contributed to 4.95 million deaths, with bacterial AMR being directly responsible for 1.27 million deaths worldwide in 2019.^1^ The misuse and overuse of antimicrobials in humans, animals, and plants are the primary factors driving the development of drug-resistant pathogens. This phenomenon jeopardizes the advancements made in modern medicine, as it complicates the treatment of infections and increases the risks associated with medical procedures.^2–4^

*Escherichia coli* and *Salmonella* are significant public health concerns worldwide as foodborne pathogens (FBPs), with a relatively high prevalence in developing countries such as Ethiopia.^5^ These bacteria are commonly found in the normal intestinal flora of various mammals, including humans and birds, and can also cause diarrhea and a range of extragastrointestinal diseases.^6^,^7^

Globally, the increase in antibiotic-resistant strains of *E. coli* and *Salmonella* is a growing concern in both human and veterinary medicine. The extensive use of antimicrobials in livestock, especially in food-producing animals, is believed to be a principal contributing factor to the development of nontyphoidal *Salmonella* (NTS) and *E. coli* strains with reduced susceptibility to different antimicrobial agents. Factors such as climate change may also play a significant role in promoting the emergence of antimicrobial-resistant pathogens.^4^,^8^ Furthermore, the rapid evolution and spread of multidrug resistant bacteria principally occur through mutation and acquisition of antimicrobial resistance genes (ARGs) via horizontal gene transfer, with interactions among humans, animals, and the environment playing a direct or indirect role in this process.^2^,^9^,^10^

In Ethiopia, the prevalence of AMR among bacteria, including *E. coli* and *Salmonella* isolates from various sources, is also rapidly increasing. Pathogens of medical importance have shown resistance rates ranging from 30% to 85% to key antimicrobial agents outlined in the Ethiopian Standard Treatment Guidelines^11^ with some reaching 100% resistance to specific drugs.^12^ Studies have shown that the pooled prevalence of bacterial AMR in food-producing animals is 20%. The overall multidrug resistance (MDR) prevalence among bacteria is 74%, with rates of 81% and 77% for *Salmonella* spp. and *E. coli*, respectively.^13^ These prevalences could be even higher because of incorrect and inappropriate use of antimicrobials and inadequate surveillance in the country.^12^ In addition to AMR concerns, the burden of pathogenic *E. coli* and *Salmonella* as FBPs is particularly high in Ethiopia. These bacteria can contaminate food products during processing and handling and pose a public health risk^5^ but are underreported due to inadequate laboratory and other capacities.

A shift from traditional detection methods to modern diagnostic technologies capable of analyzing the genomic profiles of pathogens has enhanced the field of diagnostic laboratories, including microbiology, to provide better realistic results. The identification of specific resistance genes and other associated genetic changes provides greater insight into the evolution and spread of drug-resistant strains within and between different sources (eg, humans, animals, and the environment), leading to a more holistic and accurate assessment of resistance profiles.

However, there is a scarcity of comprehensive studies that have generated data on the AMR profiles of FBPs using advanced diagnostic technologies, such as whole genome sequencing (WGS) in Ethiopia. Previous studies conducted in the country have focused on reporting the prevalence and phenotypic AMR patterns of these pathogens separately in human, animal, or food samples using routine laboratory methods. Hence, antimicrobial resistance profiling, including phenotypic resistance prediction of FBPs in a one health study approach, is important for the development of integrated targeted interventions and better strategies to combat the spread of AMR in the country. Therefore, the objective of this study was to predict and characterize the antimicrobial resistance profiles of diarrheagenic *E. coli* and nontyphoidal *Salmonella* strains isolated from children with diarrhea and their exposures, including caretakers, animal, food, and environmental samples in Ethiopia, using whole genome sequencing.

### Sample Type and Collection Procedures

The diarrheagenic *E. coli* (DEC) and nontyphoidal *Salmonella* (NTS) isolates analyzed in this study were obtained from children under 5 years of age (UFC) with diarrhea, their caretakers, and various environmental samples collected through case-based tracking between November 2021 and January 2023 in Harar town and Kersa district, eastern Ethiopia. Initially, the identification of children with diarrhea was performed at healthcare facilities. Following the screening of eligible UFC cases, their possible contacts, including domestic animals, food, and environmental samples (water and wastewater), were traced and collected from each child’s exposure environment. The study methods and sample collection procedures for stool and environmental samples are detailed in our recent articles.^14^,^15^

### Sample Preparation, Bacterial Isolation and Identification

Stool and environmental samples were processed following a standard operating procedure (SOP) created for the FOCAL project, with minor modifications to the existing protocols.^16^,^17^ The environmental samples were prepared using a 1:9 (v/v) ratio of sample-to-buffered peptone water (BPW) broth and a 1:1 ratio for the nonturbid water samples. Meat samples were prepared in a 1:3 ratio of sample-to-modified tryptic soy broth (mTSB), as described earlier.^15^

The isolation and identification of NTS and DEC were conducted using culture and biochemical tests, as previously described.^14–16^ This protocol involves the use of pre-enrichment, selective and differential media and biochemical confirmation tests. All the samples were incubated at 37°C for 24 hours. However, the meat samples were prepared in mTSB and incubated at 37°C for 4 hours and then at 42°C for 20 ± 1 hours. Following the incubation period, aliquots from the pre-enrichment mixture were streaked on xylose lysine deoxycholate (XLD), Hektoen Enteric Agar (HE), and MacConkey agar (MAC). At the same time, 1 mL and 0.1 mL of the broth suspensions were incubated with tetrathionate and Rappaport vassiliadis soya broths and plated on both XLD and HE for *Salmonella* isolation. Characteristic colonies were picked and subjected to Gram staining. The *E. coli* suspected gram-negative rod-shaped colonies on the MAC were further transferred to Levine’s eosin-methylene blue (EMB) agar.

Characteristic colonies of *Salmonella* on XLD and/or HE and *E. coli* on EMB plates were then identified using a series of biochemical tests, including triple sugar iron and lysine iron agar, urease, and IMViC (Indole (I), methyl red (M), Voges proskauer (Vi), and citrate (C)) media, and the results were interpreted according to Andrews et al.^17^ Biochemically confirmed *E. coli* isolates were then further plated on sorbitol MacConkey agar, and only biochemically confirmed, nonsorbitol fermenter isolates were considered pathogenic *E. coli* or DEC.^14^

### DNA Extraction and Whole Genome Sequencing

Genomic DNA extraction and sequencing were carried out at Kilimanjaro Clinical Research Institute, Biotechnology Laboratory (KCRI_BL) in Moshi, Tanzania. DNA was extracted from a fresh pure colony using Quick-DNA*™* Fungal/Bacterial Miniprep Kit according to the manufacturer’s instructions.^18^ The quantity and quality of the DNA were assessed before library preparation using a Qubit 2 fluorometer. Only samples with an absorbance A260/A280 ratio of 1.8 or above were considered for further analysis.

Next generation sequencing (NGS) libraries were prepared using the Illumina DNA library Prep (Illumina Inc., USA) as previously described^19^ and sequenced on an Illumina NextSeq 550 instrument with 2 × 150 bp paired-end reads.

### Bioinformatics Analysis

Various bioinformatics tools, such as *FastT*QC, *ResFinder*, and *PointFinder*, were employed for the genomic analysis of the raw reads. The paired-end WGS reads of each isolate were preprocessed using an in-house quality control (QC) and assembly pipeline using BBDuk^20^ and *Fast*QC^21^ for adapter trimming and quality checking.

### Species Determination

Species determination was conducted by uploading each paired-end sequence to the *SpeciesFinder* 2.0 program (https://cge.food.dtu.dk/services/SpeciesFinder/) and selecting Illumina paired-end reads from the tool’s choices. The sequences were also aligned against redundant databases using the kmer alignment (KMA) tool^22^ accessed from the Center for Genomic Epidemiology (CGE) page at https://cge.food.dtu.dk/services/KmerFinder/ to determine the isolate species.

### Genomic Antibiotic Resistance Analysis

In the present study, WGS-based phenotypic AMR prediction was performed according to Bortolaia et al^23^ and Clausen et al^24^ using the *ResFinder* tool 4.4 (http://genepi.food.dtu.dk/resfinder) and *KmerResistance* 2.2 tools accessed from https://cge.food.dtu.dk/services/KmerResistance/. The analysis included the assessment of acquired ARGs and point mutations of 57 NTS and 50 DEC isolates, with default settings of the tool, such as a 90% identity threshold and a minimum 60% matching alignment or gene length.

In general, using the *ResFinder* tool, we detected ARGs, antimicrobial classes, and phenotypes (drugs) associated with identified ARGs. Moreover, *PointFinder*, an extension of the *ResFinder* tool, identified point mutations linked to AMR. *Salmonella* serotypes were determined using *SeqSero*.^25^ The raw sequences of the isolates analyzed in this study have been submitted to the European Nucleotide Archive (ENA) under accession number PRJEB73590.

Furthermore, the prevalence of AMR was calculated on the basis of the total number of isolates analyzed: 50 for DEC and 57 for NTS. Upon the identification of ARGs conferring resistance to three or more phenotypes (antimicrobial drugs), we considered it a predictor of MDR.

## Results

The current study employed whole genome sequencing (WGS) to predict the phenotypic antimicrobial resistance (AMR) of nontyphoidal *Salmonella* (NTS) and diarrheagenic *E. coli* (DEC) isolates obtained from various sources in Ethiopia. During the study, a total of 783 samples were analyzed in the laboratory. From these samples, 72 NTS and 58 DEC isolates were initially identified. However, only 57 NTS and 50 DEC isolates were analyzed for their genotypic AMR profiles, as they had good sequence quality.

In each sequenced NTS sample, at least one acquired ARG was identified, with 22 distinct ARGs that demonstrated resistance to 10 different classes of antimicrobials (Table 1). In the case of the DEC strain, 78% (39/50) of the isolates were positive for at least one ARG, and a total of 40 ARGs conferring resistance to 11 antimicrobial classes were detected (Table 2).

**Table 1:** Prevalence of Antimicrobial Resistance Genes (ARGs) and Their Respective Predicted Phenotypic Resistance in Nontyphoidal *Salmonella* (NTS) Strains in Ethiopia (n = 57)

Antimicrobial Class	Phenotype (Antimicrobial Agent)	ARG	Isolate (%)
**Aminoglycoside**	AMK, TOB	*aac*(6′)-Iaa	57 (100)
	STR	*aph*(3′)-Ib	34 (59.65)
		*aph*(6)-Id	34 (59.65)
**Aminocyclitol, aminoglycoside**	SPC, STR	*aad*A1	1 (1.75)
**β-lactam**	FEP, AMP, CTX, CTZ, PIP, AMX, CRO, TIC, ATM	*bla*_CTX-M-15_	2 (3.51)
	AMP, CEF, PIP, AMX, TIC	*bla*_TEM-1B_	10 (17.54)
		*bla*_TEM-1D_	2 (3.51)
	AMP, PEN, PIP, AMX	*bla*_Z_	1 (1.75)
**Folate pathway antagonist**	TMP	*dfr*A1	1 (1.75)
		*dfr*A12	1 (1.75)
		*dfr*A14	3 (5.26)
		*dfr*A8	1 (1.75)
		*dfr*G	1 (1.75)
	SMX	*sul*2	36 (63.16)
**Fosfomycin**	FOS	*fos*A7	3 (5.26)
		*fos*B	1 (1.75)
**Macrolide**	TEL, SPI	*mph*(A)	1 (1.75)
**Macrolide, streptogramin b**	AZI, VGS, QUP, ERY, TEL, PRS	*msr*(E)	1 (1.75)
**Quinolone**	CIP	*qnr*S1	2 (3.51)
**Peroxide**	H2O2	*sit*ABCD	3 (5.26)
**Tetracycline**	DOX, TET	*tet*(A)	38 (66.67)
		*tet*(K)	1 (1.75)

**Abbreviations**: TOB, Tobramycin; AMK, Amikacin; SPC, Spectinomycin; STR, Streptomycin; CTZ, Ceftazidime; ATM, Aztreonam; CRO, Ceftriaxone; FEP, Cefepime; CTX, Cefotaxime; TIC, Ticarcillin; PIP, Piperacillin; AMP, Ampicillin; AMX, Amoxicillin; TMP, Trimethoprim; QUP, Quinupristin; PRS, Pristinamycin; VGS, Vancomycin; ERY, Erythromycin; TEL, Telithromycin; AZI, Azithromycin; SPI, Spiramycin; CIP, Ciprofloxacin; H2O2, Hydrogen peroxide; SMX, Sulfamethoxazole; TET, Tetracycline; DOX, Doxycycline; CEF, Cephalothin; PEN, Penicillin; FOS, Fosfomycin.

**Table 2:** Prevalence of Antimicrobial Resistance Genes (ARGs) and Their Respective Predicted Phenotypic Resistance in Diarrheagenic *E. Coli* (DEC) Isolates in Ethiopia (n = 50)

Antimicrobial Class	Phenotype (Antimicrobial Agent)	ARG	Isolate (%)
**Aminoglycoside**	SIS, DBK, GEN, APR, TOB and NET	*aac*(3)-IId	1 (2)
	TOB and GEN	*aac*(3)-IIa	1 (2)
		*aac*(6′)-Iaa	3 (6)
	STR and TOB	*aph*(6)-Id	26 (52)
	STR	*ant*(3″)-Ia	2 (4)
		*aph*(3′)-Ib	27 (54)
	STR	*aad*A1	9 (18)
**Aminocyclitol**	SPC		
	SPC	*aad*A2	4 (8)
**Aminoglycoside**	STR		
**Aminocyclitol**	SPC	*aad*A24	1 (2)
**Aminoglycoside**	STR		
	STR	*aad*A5	2 (4)
**Aminocyclitol**	SPC		
**Beta-lactam (β-lactam)**	ATM, AMX, CTZ, PIP, FEP, AMP, TIC, CTX and CRO	*bla*_CTX-M-14b_	1 (2)
		*bla*_CTX-M-15_	9 (18)
		*bla*_CTX-M-27_	3 (6)
		*bla*_CTX-M-69_	1 (2)
		*bla*_CTX-M-71_	1 (2)
		*bla*_KLUC-2_	1 (2)
	PIP+TZP, AMX+CLA, AMP+CLA, AMP, AMX, and FEP	*bla*_OXA-1_	1 (2)
	FEP, CRO, CTZ, ATM, AMP, TIC, PIP and AMX	*bla*_TEM-169_	1 (2)
	AMX, AMP, CEF, PIP and TIC	*bla*_TEM-1B_	22 (44)
	CEF	*bla*_TEM-1D_	1 (2)
	AMP+CLA, AMX+CLA, PIP+TZP, TIC+CLA, AMP, TIC, PIP and AMX	*bla*_TEM-35_	1 (2)
**Folate pathway antagonist**	TMP	*dfr*A1	8 (16)
		*dfr*A12	4 (8)
		*dfr*A14	8 (16)
		*dfr*A15	3 (6)
		*dfr*A17	2 (4)
		*dfr*A8	7 (14)
	SMX	*sul*1	8 (16)
	SMX and CIP	*sul*2	26 (52)
**Lincosamide**	CLI and LCM	*erm*(B)	1 (2)
**Streptogramin B**	QUP, PRS and VGS		
**Macrolide**	ERY		
	TEL, ERY, AZI and SPI	*mph*(A)	11 (22)
**Quinolone**	CIP and NAL	*par*C	1 (2)
		*gyr*A	15 (30)
	CIP	*qep*A4	2 (4)
		*qnr*S1	6 (12)
**Tetracycline**	TET, and DOX	*tet*(A)	17 (34)
	DOX, MIN and TET	*tet*(B)	18 (36)
	DOX andTET	*tet*(D)	2 (4)
**Amphenicol**	CHL	*cat*A1	9 (18)
**Peroxide**	H2O2	*sit*ABCD	20 (40)
**Negative for ARGs**			11 (22)

**Abbreviations**: GEN, Gentamicin; SIS, Sisomicin; DBK, dibekacin; APR, Apramycin; NET, Netilmicin; CLA, Clavulanic acid; IP, Imipenem; CHL, Chloramphenicol; CLI, Clindamycin; LCM, Linezolid; MIN, Minocycline; TZP, tazobactam; NAL, Nalidixic acid.

Notably, the *aac*(6′)-Iaa gene, which acts against aminoglycosides, was detected in all analyzed NTS strains, indicating that all the isolates were resistant to tobramycin and amikacin. The ARGs *tet*(A),* sul*2,* aph*(6)-*Id, aph*(3″)-Ib, and *bla_TEM-1B_* were also frequently detected in NTS samples, with frequency rates of 66.7% (n = 38), 63.2% (n = 36), 59.7% (n = 34), 59.7% (n = 34), and 17.5% (n=10) resistance to their corresponding antimicrobial classes, respectively. In contrast, genes such as *aad*A1, *bla*_Z_, *dfr*A1, *dfr*A12,* dfr*A8, *dfr*G, *fos*B, *mph*(A), *msr*(E), and *tet*(K) were detected less frequently, with each being identified at a rate of only 1.8% (Table 1).

Among the 40 ARGs identified in DEC strains, the most commonly detected ARGs were those conferring resistance to aminoglycosides, specifically *aph*(3′)-Ib (n = 27, 54%) and *aph*(6)-Id (n = 26, 52%), followed by the folate pathway inhibitor *sul*2 (n = 26, 52%). Other frequently identified ARGs were *bla_TEM_*_−1B_ (n = 22, 44%), *sit*ABCD (n = 20, 40%), *tet*(B) (n = 18, 36%), *tet*(A) (n = 17, 34%), and *gyr*A (n = 15, 30%), indicating the prevalence of DEC isolates resistant to β-lactams, peroxides, tetracycline, and quinolones, respectively. On the other hand, the prevalence of ARGs encoding reduced susceptibility to ciprofloxacin and nalidixic acid (*par*C) and resistance to streptomycin, spectinomycin (*aad*A1 and *aad*A24), and cefepime (*bla_TEM_*_−1D_) was only 2% (1/50) (Table 2).

**Figure 1:**
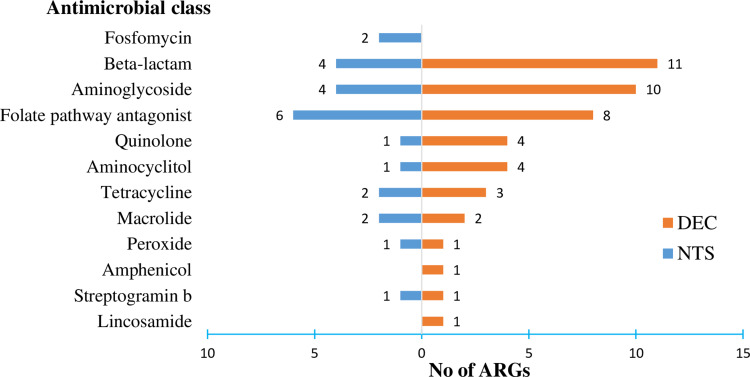
Frequencies of antimicrobial resistance genes (ARGs) detected per antimicrobial class in nontyphoidal *Salmonella* (NTS) and diarrheagenic *E. coli* (DEC) strains.

In DEC samples, the number of ARGs identified per antimicrobial class varied from one (for example, *cat*A1 and *erm*(B)) to 11 (for β-lactam class-inhibiting genes), whereas the corresponding range in NTS samples was from 1 to 6 (Figure 1).

The *ResFinder* tool also identified ARGs that inhibite the action of multiple phenotypes, indicating the existence of multidrug resistant DEC and NTS strains in the study area. Specifically, *bla*_TEM-1B_ alone predicted that 17.5% of the NTS strains were multidrug resistant to β-lactam drugs such as ampicillin, cephalothin, piperacillin, amoxicillin, and ticarcillin (Table 3). Moreover, 33.3% of the DEC isolates were predicted to be resistant to 9 antimicrobial drugs, including amoxicillin, ampicillin, aztreonam, ceftazidime, piperacillin, ceftriaxone, cefepime, cefotaxime, and ticarcillin, owing to the presence of the ARGs *bla_CTX_*_−M_ (*bla_CTX_*_−M-14b_, *bla_CTX_*_−M-15_, *bla_CTX_*_−M-27_, *bla_CTX_*_−M-71_, *bla_CTX_*_−M-69_). *bla_TEM_*_−169_ was also found to confer resistance to 8 of these drugs, with the exception of cefotaxime, but this ARG was detected in only one DEC isolate (Table 4). However, some ARGs conferred resistance to a single drug; for example, *sul*1 exhibited resistance against sulfamethoxazole, *cat*A1 against chloramphenicol, *qep*A4, and *qnr*S1 against ciprofloxacin, and *bla_TEM_*_−1D_ against cefepime in DEC isolates.

**Table 3:** Frequency Distribution of Antimicrobial Resistance Genes (ARGs) Detected in Nontyphoidal *Salmonella* (NTS) Strains Isolated from Various Sample Sources in Eastern Ethiopia (n = 57)

ARG	UFC (n=14)	Caretaker (n=5)	Animal (n=8)	Food (n=16)	Wastewater (n=13)	Water source (n=1)	Isolate (%)
***aac*(6′)-Iaa**	14 (100)	5 (100)	8 (100)	16 (100)	13 (100)	1 (100)	57 (100)
***aad*A1**	–	–	–	1 (6.25)	–	–	1 (1.75)
***aph*(3″)-Ib**	7 (50)	4 (80)	3 (37.5)	9 (56.25)	10 (76.92)	1 (100)	34 (59.65)
***aph*(6)-Id**	8 (57.14)	3 (60)	3 (37.5)	9 (56.25)	10 (76.92)	1 (100)	34 (59.65)
***bla*_CTX-M-15_**	–	–	–	2 (12.5)	–	–	2 (3.51)
***bla*_TEM-1B_**	4 (28.57)	–	1 (12.5)	3 (18.75)	2 (15.38)	–	10 (17.54)
***bla*_TEM-1D_**	–	–	1 (12.5)	–	1 (7.69)	–	2 (3.51)
***bla*_Z_**	–	–	–	–	1 (7.69)	–	1 (1.75)
***dfr*A1**	–	–	–	1 (6.25)	–	–	1 (1.75)
***dfr*A12**	–	–	–	1 (6.25)	–	–	1 (1.75)
***dfr*A14**	2 (14.29)	–	–	1 (6.25)	–	–	3 (5.26)
***dfr*A8**	1 (7.14)	–	–	–	–	–	1 (1.75)
***dfr*G**	–	–	–	–	1 (7.69)	–	1 (1.75)
***fos*A7**	1 (7.14)	–	1 (12.5)	–	1 (7.69)	–	3 (5.26)
***fos*B**	–	–	–	–	1 (7.69)	–	1 (1.75)
***mph*(A)**	–	–	–	–	1 (7.69)	–	1 (1.75)
***msr*(E)**	–	–	–	–	1 (7.69)	–	1 (1.75)
***qnr*S1**	1 (7.14)	–	–	1 (6.25)	–	–	2 (3.51)
***sit*ABCD**	2 (14.29)	–	1 (12.5)	–	–	–	3 (5.26)
***sul*2**	9 (64.29)	3 (60)	3 (37.5)	10 (62.5)	10 (76.92)	1 (100)	36 (63.16)
***tet*(A)**	10 (71.43)	3 (60)	4 (50)	9 (56.25)	11 (84.62)	1 (100)	38 (66.67)
***tet*(K)**	–	–	–	–	1 (7.69)	–	1 (1.75)

**Table 4:** Frequency Distribution of Antimicrobial Resistance Genes (ARGs) Detected in Diarrheagenic *E. Coli* (DEC) Isolated from Various Sample Sources in Eastern Ethiopia (n = 39)

ARGs	UFC (n=25)	Caretaker (n=6)	Animal (n=7)	Food (n=7)	Wastewater (n=4)	Water source (n=1)	Total
*aac*(*aac*(3)-IIa, *aac*(3)-IId, *aac*(6′)-Iaa)	3 (12)	1 (16.67)	–	–	1 (25)	–	5 (12.82)
*aad*(*aad*A1, *aad*A2, *aad*A24, *aad*A5)	5 (20)	3 (50)	4 (57.14)	3 (42.86)	–	–	15 (38.46)
*ant*(3″)-Ia	2 (8)	–	–	–	–	–	2 (5.13)
*aph*(*aph*(3″)-Ib, *aph*(6)-Id)	14 (56)	4 (66.67)	4 (57.14)	2 (28.57)	2 (50)	1 (100)	27 (69.23)
*bla*_CTX-M_(*bla*_CTX-M-14b,_ *bla*_CTX-M-15_, *bla*_CTX-M-27_, *bla*_CTX-M-71_, *bla*CTX-M-69)	4 (16)	2 (33.33)	3 (42.86)	2 (28.57)	2 (50)	–	13 (33.33)
*bla*_KLUC-2_	–	–	1 (14.29)	–	–	–	1 (2.56)
*bla*_OXA-1_	1 (4)	–	–	–	–	–	1 (2.56)
*bla*_TEM_(*bla*_TEM-169_, *bla_T_*_EM-1B_, *bla*_TEM-1D_, *bla*_TEM-35_)	9 (36)	4 (66.67)	4 (57.14)	4 (57.14)	1 (25)	1 (100)	23 (58.97)
*cat*A1	3 (12)	3 (50)	1 (14.29)	–	1 (25)	1 (100)	9 (23.08)
*dfr*A(*dfr*A1, *dfr*A12, *dfr*A14, *dfr*A15, *dfr*A17, *dfr*A8)	11 (44)	4 (66.67)	5 (71.43)	4 (57.14)	1 (25)	1 (100)	26 (66.67)
*erm*(B)	1 (4)	–	–	–	–	–	1 (2.56)
*gyr*A	8 (32)	3 (50)	3 (42.86)	–	1 (25)	–	15 (38.46)
*mph*(A)	5(20)	2 (33.33)	1 (14.29)	1 (14.29)	1 (25)	1 (100)	11 (28.21)
*par*C	–	–	–	–	1 (25)	–	1 (2.56)
*qep*A4	–	1 (16.67)	–	–	1 (25)	–	2 (5.13)
*qnr*S1	1 (4)	–	2 (28.57)	1 (14.29)	1 (25)	–	5 (12.82)
*sit*ABCD	13 (52)	3 (50)	3 (42.86)	–	1 (25)	–	20 (51.28)
*sul*(*sul*1, *sul*2)	12 (48)	4 (66.67)	5 (71.43)	4 (57.14)	3 (75)	1 (100)	29 (74.36)
*tet*(*tet*(A), tet(B), *tet*(D))	14 (56)	4 (66.67)	5 (71.43)	4 (57.14)	4 (100)	1 (100)	32 (82.05)
**Total positive**	**19 (76)**	**5 (83.33)**	**6 (85.71)**	**4 (57.14)**	**4 (100)**	**1 (100)**	**39 (100)**

The 57 NTS isolates analyzed for genotypic AMR profiles were obtained from various sources, including children with diarrhea (n = 14), caretakers (n = 5), animals (n = 8), food (n = 16), water (n = 1), and wastewater (n = 13) samples. As shown in Table 3, NTS isolates from wastewater contained the greatest diversity of ARGs, followed by the food and UFC samples. ARGs such as *aac*(6′)-Iaa, *aph*(3′)-Ib, *aph*(6)-Id, *sul*2, and *tet*(A) were detected across all sample types, but *dfr*A8 was identified in only the UFC sample, and *bla*_Z_, *dfr*A1, *dfr*A12, *dfr*G, *fos*B, and *mph*(A) were detected in only the wastewater samples.

Regarding sample types, genotypic antimicrobial resistance (AMR) profiling was conducted on DEC isolates obtained from UFC cases (n = 25), caretakers (n = 6), animals (n = 7), food (n = 7), water sources (n = 1), and wastewater (n = 4) samples. Among the 39 DEC samples that tested positive for ARGs, the detection frequencies of ARGs were 76%, 83.3%, 85.7%, and 57.1% for the UFC, caretaker, animal, and food isolates, respectively. All four wastewater isolates and a single water source isolate analyzed in this study tested positive for at least one ARG. The findings also revealed that across all sample categories, at least one ARG from the *dfr*A group (*dfr*A1, *dfr*A12, *dfr*A14, *dfr*A15, *dfr*A17, *dfr*A8), *tet* group (*tet*(A), *tet*(B), *tet*(D)), *sul* group (*sul*1, *sul*2), *mph*(A), *bla_TEM_* group (bla_TEM-169_, bla_TEM-1B_, bla_TEM-1D_, bla_TEM-35_), and *aph* group (*aph*(3″)-Ib, *aph*(6)-Id) that provides resistance to commonly used drugs were detected. However, some ARGs were detected only in a specific sample source (Table 4).

**Figure 2:**
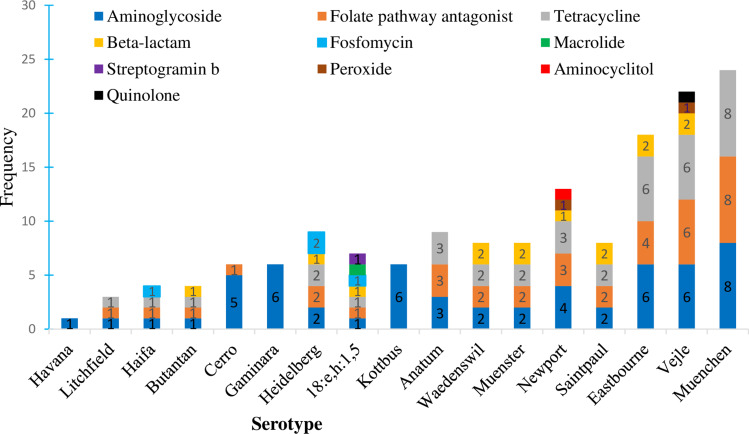
Number of antimicrobial resistance genes (ARGs) detected against different antimicrobial classes in different *Salmonella* serotypes.

Among the identified NTS serotypes, some strains had developed resistance to multiple antimicrobial classes. Notably, both S. Newport and S. Vejle were the predominant strains carrying genes conferring resistance to six antimicrobial classes and were the most frequently identified multidrug resistant serotypes. Each of the eight S. Muenchen strains identified in this study harbored genes conferring resistance to aminoglycosides, folate pathway antagonists, and tetracycline classes. Conversely, only genes associated with aminoglycoside resistance were identified in S. Gaminara, S. Kottbus, and S. Havana (Figure 2).

### Point Mutation

In addition to the acquired genes, the present study identified mutations that mediate AMR using the *PointFinder* tool. The analysis revealed 917 point mutations in NTS isolates and 1430 point mutations in DEC isolates, with the majority of these mutations being linked to nucleotide substitutions (adenine (A), thymine (T), cytosine (C), or guanine (G)) (Table 5, Supplementary data S1 and Supplementary data S2).

**Table 5:** Frequencies of Point Mutations in Genes Predicting Antimicrobial Resistance in Diarrheagenic *E. Coli* (DEC) and Nontyphoidal *Salmonella* (NTS) Isolates from Various Sources in Ethiopia

Gene/Region	Mutation Type in DEC (%)					Mutation Type in NTS (%)				
	Deletion	Insertion	Substitution	Truncation	Subtotal	Deletion	Insertion	Substitution	Truncation	Subtotal
16S_*rrs*B	–	–	100 (100)	–	100 (6.99)	–	–	–	–	–
16S_*rrs*C	1 (1.39)	2 (2.78)	69 (95.83)	–	72 (5.03)	–	–	–	–	–
16S_*rrs*D	–	–	–	–	–	23 (4.75)	30 (6.2)	431 (89.05)	–	484 (52.78)
16S_*rrs*H	2 (3.57)	–	54 (96.43)	–	56 (3.92)	–	–	–	–	–
23S	28 (3.11)	32 (3.56)	840 (93.33)	–	900 (62.94)	–	–	–	–	–
*amp*C	–	–	15 (68.18)	7 (31.82)	22 (1.54)	–	–	–	–	–
*acr*B	–	–	–	–	–	1 (0.83)	–	120 (99.17)	–	121 (13.2)
*fol*P	–	–	9 (100)	–	9 (0.63)		–	–	–	–
*gyr*A	–	–	51 (100)	–	51 (3.57)	2 (4.76)	–	39 (92.86)	1 (2.38)	42 (4.58)
*gyr*B	–	–	21 (100)	–	21 (1.47)	–	–	14 (100)	–	14 (1.53)
*par*C	–	–	60 (100)	–	60 (4.2)	1 (0.84)	–	117 (98.32)	1 (0.84)	119 (12.98)
*par*E	–	–	23 (100)	–	23 (1.61)	–	–		–	
*pmr*A	–	–	9 (100)	–	9 (0.63)	–	–	21 (100)	–	21 (2.29)
*pmr*B	–	–	44 (100)	–	44 (3.08)	–	–	116 (100)	–	116 (12.65)
*rpo*B	–	–	62 (98.41)	1 (1.59)	63 (4.41)	–	–	–	–	–
**Total**	**31 (2.17)**	**34 (2.38)**	**1357 (94.9)**	**8 (0.56)**	**1430 (100)**	**27 (2.94)**	**30 (3.27)**	**858 (93.57)**	**2 (0.22)**	**917 (100)**

Point mutations were most commonly observed in the *rrs* genes, followed by *acr*B and *rpo*B gene mutations, at rates of 13.2% (121 out of 917) in NTS and 4.4% (63 out of 1430) in DEC. In the DEC strains, the predominant amino acid substitution linked to *rpo*B point mutations was the replacement of threonine with isoleucine at position 595 (ie, *rpo*B:p.T595I) (Supplementary S1). In contrast, *acr*B:p.F28L and *acr*B:p.L40P mutations were detected in every NTS strain with the substitution of phenylalanine by leucine at position 28 and leucine by proline at position 40 (Supplementary S2). Moreover, the study identified other point mutations in the genes *gyr*A, *gyr*B, *par*C, *pmr*A, and *pmr*B in both of these pathogens and *amp*C, *fol*P, and *par*E exclusively in DEC isolates that resulted in AMRs (Table 5).

## Discussion

The increasing prevalence of AMR among bacterial pathogens, such as *Salmonella* and *E. coli*, is a significant risk to public health, particularly in developing countries, such as Ethiopia.^3^,^13^,^26^,^27^ The results of the present study indicated that every sequenced NTS strain and 78% of the *E. coli* isolates harbored at least one ARG. Resistance was detected across all sample categories in both pathogens, highlighting the importance of AMR in these pathogens regardless of the sample source. This widespread occurrence could also be attributed to the involvement of many actors in the dissemination of drug-resistant pathogens. Human, animal, and environmental components interact with and either directly or indirectly contribute to the spread of antimicrobial resistant strains.^10^

Similarly, a study conducted on captive bears in China identified ARGs in 88% of *E. coli* strains.^28^ However, studies conducted in Ethiopia^3^ and elsewhere^29^ have identified at least one ARG in every *E. coli* strain analyzed. Unlike the current study, a study conducted in Cambodia predicted phenotypic AMR in only 53% of NTS isolates.^30^

The prediction of phenotypic resistance in every NTS strain may also be attributed to a mutation in *acr*B, which can expel multiple classes of antibiotics, including macrolides, β-lactams, quinolones, rifamycins, tetracycline, etc.^31^ A point mutation in *acrB* was observed in all the analyzed NTS isolates. Specifically, point mutations *acr*B:p.F28L and *acr*B:p.L40P, which led to the replacement of phenylalanine with leucine at position 28 and leucine with proline at position 48, may contribute to the development of widespread resistance in NTS isolates. Mutations in *acr*B provide the principal AMR efflux function in *Salmonella enterica*, such as *S*. Typhimurium, and confer resistance to antibiotics, such as cefotaxime and azithromycin.^32^,^33^ However, a previous study reported *acr*A, *acr*B, *acr*D, and *tol*C in 68.8% of NTS.^34^

Nucleotide substitutions (A, T, C, or G) were the most frequently detected mutations, accounting for 94.9% of DEC isolates and 93.6% of NTS isolates. Variations in mutation frequency were also observed among the isolates, with some isolates exhibiting multiple mutations. A total of 917 and 1430 point mutations were observed across all sequenced NTS and DEC isolates, respectively, which can lead to changes in the structure or function of the proteins they encode.^35^,^36^

This study identified diverse ARGs that confer resistance to 11 and 10 different classes of antimicrobials in DEC and NTS, respectively. The majority of these ARGs have also been described in several studies, including one health study^37^ and other studies in humans and animals.^28^,^29^,^38–40^ However, a study conducted in Malaysia identified only 15 ARGs.^41^ In the NTS, the number of ARGs identified among the antimicrobial classes ranged from 1 to 6, whereas in the case of DEC, it reached 11. *E. coli* also exhibited a wider array of ARGs than NTS strains did, possibly because of its frequent exposure to antibiotics for different purposes in both human and veterinary medicine.^42^ The differences in the number of identified ARGs and their distributions among NTS and DEC strains in various studies could be attributed to variations in epidemiological factors, analytical methods, study populations (hosts), sample sizes, study periods, and so on.^43^

In some strains, *ResFinder* analysis predicted resistance to multiple phenotypes, indicating the presence of multidrug resistant DEC and NTS strains in the study area. For instance, *bla*_CTX-M-15_ alone predicted that 18% of DEC strains and 3.5% of NTS strains were resistant to up to nine phenotypes, including amoxicillin, ampicillin, aztreonam, ceftazidime, piperacillin, ceftriaxone, cefepime, cefotaxime, and ticarcillin. Previous studies have documented similar MDR profiles for these pathogens.^38^,^41^,^44^,^45^

Similarly, S. Newport and S. Vejle were predicted to be resistant to six antimicrobial classes, and each identified S. Muenchen strain carried at least one gene that reduced susceptibility to aminoglycosides, folate inhibitors, and tetracycline classes. This implies the existence of MDR in many NTS serotypes in Ethiopia, possibly stemming from improper use of antibiotics across different sectors. The emergence and spread of multidrug resistant *Salmonella* serotypes, including *S*. Muenchen^46^,^47^ and *S*. Newport,^40^,^48^ have been reported in previous studies. However, serotypes such as *S*. Gaminara, *S*. Kottbus, and *S*. Havana, were found to have ARGs conferring resistance to a single antimicrobial class.

Tetracycline class-encoding resistance genes (*tet*) were the most frequently detected ARGs in *E. coli* and the second most frequently detected ARGs in NTS isolates. Our findings are in partial agreement with those of a previous study in which genes conferring resistance to tetracycline were the most prevalent at a rate of 76.8% in *E. coli* isolates.^28^ However, the prevalence of ARGs associated with tetracycline in NTS strains was lower than that reported in a study conducted in animals (86.4%) by Kahsay et al.^27^ Tetracycline drugs are among the most widely used antimicrobials in human and veterinary medicine. However, increased resistance to doxycycline and tetracycline, primarily attributed to *tet*(B) in DEC and associated with *tet*(A) in NTS strains, suggests the need for alternative drugs. A recent study conducted in China reported that only 17.3% of NTS isolates had *tet*(A).^49^ Moreover, the detection of *tet*(D) in DEC samples and *tet*(K) in NTS strains demonstrated the involvement of multiple ARGs.

Previous studies have reported that *tet*(A) is the most common tetracycline resistance gene.^3^,^37^,^43^,^50^,^51^ However, *tet*(K) has not been reported in NTS strains in several previous studies,^43^,^51^,^52^ and no tetracycline resistance predicting genes have been detected in *E. coli* isolated from farmed minks in China.^53^

The higher prevalence of tetracycline resistance predicting genes in *E. coli* and NTS strains found in wastewater can be attributed to the diverse sources of waste analyzed in this study, including abattoirs, hotels, restaurants, households, and animal farms. Wastes from such diverse sources are likely to be contaminated with commonly used antimicrobials, such as tetracycline, and antimicrobial-resistant organisms. Hence, pathogens can change their mechanisms of action to survive in the presence of these drugs. These findings are consistent with those of a previous study conducted in Ethiopia.^3^,^40^

This study also revealed a wide range of ARGs (up to 11) associated with β-lactam activity in DEC strains. These genes predicting resistance to β-lactams were also the second most commonly detected genes in DEC, with a frequency of 74.4% (29/39) and in 24.6% (14/57) of NTS isolates. Except for *bla_TEM_*_−1D_ in DEC isolates, all β-lactam-linked ARGs identified in this study were predicted to confer resistance to more than three antibiotics, demonstrating the presence of multidrug resistant *E. coli* and NTS strains, as reported earlier.^50^ The rate of β-lactam resistance genes in DEC partially aligns with a report by Sonda et al,^50^ who reported a prevalence of 70%. Among these genes, *bla_TEM_*_−1B_ was identified as the most prevalent (44%), followed by *bla_CTX_*_−M-15_ (18%). Similarly, a study conducted in Denmark identified *bla_TEM_*_−1B_ as the predominant gene.^39^ In contrast, previous studies identified *bla_OX_*_−A-1_^50^ and *bla_CTX_*_−M-15_^54^ as the most common β-lactam ARGs in *E. coli*.

On the other hand, the ARG *bla_CTX-_*_M-15_ was detected in only 3.5% of the NTS isolates, which was lower than that reported in a previous study.^51^ The current ARG analysis also predicted phenotypic resistance to β-lactam drugs for several NTS serotypes. Despite the limited number of studies on the genotypic AMR profile of NTS in Ethiopia, phenotypic analyses have shown the presence of many NTS serotypes that are resistant to β-lactam drugs.^13^,^27^

Extended-spectrum β-lactamase genes, such as *bla_CTX-_*_M-15,_ confer resistance to third-generation cephalosporins, including cefotaxime, ceftriaxone, and ceftazidime, as well as fourth-generation cefepime, and worsen the treatment outcomes of these widely used drugs in humans for the treatment of gram-negative bacterial infection.^51^,^55^ On the other hand, gram-negative bacteria often have a naturally occurring β-lactamase enzyme, possibly due to the selective pressure exerted by β-lactam-producing soil organisms in the environment.^56^

Moreover, the production of *amp*C β-lactamases by bacteria poses a public health concern in the context of MDR.^57^ Similarly, our study revealed chromosomal point mutations in the *amp*C promoter region of *E. coli*, such as substitutions of cytosine with thymine at position 1, a guanine with adenine at position 18, and substitutions of the amino acid valine by phenylalanine at position 13, with arginine replaced by a stop codon at position 24 (*). Previous studies reported similar base pair (bp) and amino acid substitutions in the *amp*C promoter region of *E. coli* at different loci.^58^,^59^ Mutations in the *amp*C promoter region trigger hyperproduction and overexpression of the typically low constitutively expressed *amp*C, resulting in resistance against β-lactam drugs, including cephalosporins.^57^,^58^

Bacteria typically develop resistance to sulfonamides through both gene mutation and gene substitution strategies. Mutation of the *fol*P gene of dihydropteroate synthetase (DHPS) on the chromosome results in drug resistance, whereas gene substitution involves the acquisition of the sulphonamide resistance genes *sul*1, *sul*2, and *sul*3.^60^ Similarly, the plasmid-acquired genes *sul*1 and *sul*2 and chromosomal point mutations of *folP* conferring resistance to the folate pathway antagonist class were identified in DEC isolates in this study. DHPS is a target of sulfa drugs.^61^

Among the NTS strains, only *sul*2 was identified as conferring resistance to sulfamethoxazole and was detected at a prevalence of 63.2%. This gene predicted 76.9% and 64.3% phenotypic resistance in NTS isolates from wastewater and children, respectively, indicating widespread resistance to sulfonamide. Other studies have also documented this gene in the NTS.^43^,^51^ However, a study conducted in China reported *sul*2 only at a prevalence of 58.8%.^49^

The *sul* (*sul*1 and *sul*2) genes were detected in 74.4% (29/39) of the DEC isolates, with a relatively lower occurrence in food isolates. However, a study conducted in China reported frequency rates of 90% and 88.6% for *E. coli* isolated from *P. vannamei* and pork samples, respectively.^62^ Furthermore, the occurrence of *sul*2 was higher in animal and human isolates, indicating the spread of sulfamethoxazole and ciprofloxacin resistant *E. coli* strains among human and animal species. These two drugs are among the most widely used drugs in human medicine in Ethiopia. Similar to this study, a prior study identified *sul* genes in both diarrheic and asymptomatic human isolates,^63^ demonstrating the development, spread, and carriage of ARGs in the guts of asymptomatic individuals. However, *sul*2 has not been reported in *E. coli* strains isolated from vegetable farms in Ethiopia.^3^

Similar to previous studies, our study identified other folate inhibitor gene*s, dfr*A1, *dfr*A12, *dfr*A14, *dfr*A15, *dfr*A17, and *dfr*A8, in DEC samples^50^,^64^ as well as *dfr*A1, *dfrA*12, *dfr*A14, *dfr*A8, and *dfrG*, in NTS samples.^65^,^66^ These genes were detected in 64% and 12.3% of the DEC and NTS isolates, respectively, indicating phenotypic resistance to trimethoprim. Trimethoprim is an antibiotic that inhibits dihydrofolate reductase (DHFR) and prevents the production of tetrahydrofolate (THF), which is essential for bacterial DNA synthesis. Interestingly, *dfr*A genes were detected in *E. coli* strains from all sample types and in NTS from children with diarrhea and food samples. Humans, animals, plants, food, and the environment play principal roles in the spread of AMR.^37^ Nevertheless, the current findings suggest that trimethoprim is a better drug than sulfamethoxazole for treating DEC- and NTS-associated infections in our study area.

All the sequenced NTS isolates tested positive for the *aac*(6′)-Iaa gene, demonstrating a complete diminishment in the efficacy of tobramycin and amikacin in our study area. This finding is consistent with those of previous studies.^52^,^67^,^68^ Moreover, 59.7% of the NTS isolates were resistant to streptomycin, as predicted by *aph*(3′)-Ib and *aph*(6)-Id′. This resistance rate surpasses the 10% reported in a study from Mexico for *aph* genes.^68^ Conversely, resistance to both spectinomycin and streptomycin, which occurs through adenylyltransferase (*aad*A1) (1.8%), was lower than that reported in a previous study.^69^

On the other hand, resistance to aminoglycosides was predicted in 64% (32/50) of the DEC samples. The *aph*[*aph*(3′)-Ib, and *aph*(6)-Id′], *aad*[aadA1, *aad*A2, *aad*A24, and *aad*A5], *aac*[*aac*(3)-IId, *aac*(3)-IIa, and *aac*(6′)-Iaa], and *ant*(3″)-Ia genes contributed 69.2% (27/39), 38.5%, 12.8%, and 5.1% rates, respectively, to the development of resistance to aminoglycosides in DEC samples. Several studies have identified these genes in different populations at varying rates.^3^,^37^,^50^,^70^,^71^ However, a recent study conducted in Ethiopia reported a high prevalence of *aac*(3)–IV (86.6%) in *E. coli* isolates from soil.^3^ Factors such as sample type, epidemiology, and detection methods can contribute to variations in the prevalence of ARGs.

Bacterial resistance to fluoroquinolones is usually mediated by mutations in the DNA gyrase and topoisomerase IV genes, as well as through active efflux mechanisms. The *gyr*A and *gyr*B genes encode DNA gyrases, whereas *parC* and *par*E encode topoisomerase IV.^72^,^73^ Mutations in these genes inhibit quinolone binding and facilitate bacterial DNA replication, leading to high-level resistance. Additionally, the upregulation of efflux pumps and plasmid-borne genes, such as *qnr, qep*A, and *aac*(6′)-*Ib-cr*, can contribute to resistance.^74^,^75^ Similarly, the present study identified point mutations associated with these genes.

In this study, the *gyr*A point mutation was detected in 38% (19/50) of the DEC isolates and 45.6% (26/57) of the NTS isolates. Additionally, the acquired *gyr*A gene was detected in DEC isolates from UFC, caretaker, animal, and wastewater, demonstrating phenotypic resistance to ciprofloxacin and nalidixic acid antimicrobial agents in these isolates.

Mutations in the quinolone-resistance determining region (QRDR) of the *gyr*A gene at codons 83 and 87 are known to be crucial in the emergence of DNA gyrase-associated mutations.^76^ However, the present study identified *gyr*A mutations at different nucleotide positions other than positions 83 and 87. Other previous studies have reported the importance of point mutations outside the QRDR in conferring quinolone resistance.^76^,^77^

Among the *gyr*A gene mutations, *gyr*A:p.D759E and *gyr*A:p.A873V were the most frequently observed mutations in the NTS samples, resulting in the substitution of aspartic acid with glutamic acid at position 759 and alanine with valine at position 873. According to Rodrigues et al,^78^ amino acid substitutions alter housekeeping genes, such as DNA gyrase (*gyr*A and *gyr*B) and topoisomerase IV (*par*C and *par*E), leading to reduced susceptibility to quinolone binding.

A truncation mutation (*gyr*A:p.L134*) was detected in one NTS isolate, resulting in the replacement of leucine with a premature stop codon at position 134. Emerging *Salmonella* strains in sub-Saharan Africa have undergone extensive gene deletions and truncations. Owing to adaptive pressures, traits that are detrimental to particular environmental conditions can be inactivated to gain an immediate adaptive advantage and ensure survival.^36^,^79^

Consistent with a study conducted in Tanzania,^50^ the detection of mutations in the *gyr*A or *par*C genes that were linked to ciprofloxacin may result in a decrease in the effectiveness of this widely prescribed drug in Ethiopian healthcare system. In the present analysis, the *qnrS*1 gene was detected in 3.5% of the NTS isolates, representing the only plasmid-based ARG identified that predicts resistance to the quinolone class among NTS isolates. A study conducted in Korea also identified *qnrS* at a rate of only 0.5% in NTS samples.^80^

Furthermore, resistance to quinolone agents in DEC isolates was predicted by acquired genes such as *gyr*A, *par*C, *qep*A4, and *qnr*S1 at rates of 38.5%, 2.6%, 5.1%, and 12.8%, respectively. These findings indicate the development of diverse ARGs in certain strains of the analyzed bacterial pathogens, potentially leading to a decreased susceptibility to quinolone drugs, including ciprofloxacin. However, the level of resistance to ciprofloxacin observed in this study for both pathogens was not higher than the corresponding levels for the aminoglycoside, tetracycline, and β-lactam antimicrobial classes. It has been reported that *qnr* genes offer low resistance to quinolones in *Enterobacteriaceae*, but their multi-resistance dimensions make them highly important.^81^

Attributing to the current findings, previous studies have identified ARGs conferring resistance to lincosamide and streptogramin b (*erm*(B)),^37^,^82^ macrolides (*erm*(B) and *mph*(A)),^50^ amphenicol (*cat*A1),^37^,^50^ and peroxide (*sit*ABCD).^83^ The *sit*ABCD gene, which is found in many pathogenic enterobacteria, including *E. coli, Shigella*, and *Salmonella*, is involved in manganese and iron transportation and contributes to resistance against hydrogen peroxide,^83^,^84^ which is commonly used as a disinfectant and cleaner in many laboratories and healthcare facilities in Ethiopia.

RNA molecules are crucial for ribosomal function, particularly at key sites, such as the decoding center in 16S rRNA and the peptidyl transferase center of 23S rRNA. Consequently, many antibiotics target these RNA sites, and several mutations confer resistance to these antibiotics.^85^ Aminoglycoside agents are positively charged oligosaccharides that specifically target bacterial ribosomes and inhibit protein synthesis by binding to the 16S ribosomal subunit, ultimately leading to bacterial cell death. Point mutations in 16S rRNA and 23S rRNA genes may also be linked to MDR, as previously reported for spectinomycin.^31^,^86^ Similarly, the mutation of the 23S and 16S_*rrs* genes detected in the present study could contribute to the development of resistance to aminoglycosides, such as tobramycin, amikacin, streptomycin, spectinomycin, and gentamicin. Resistance linked to *rrs* gene mutations has also been previously described for bacteria other than DEC and NTS isolates.^87^

The prevalence of point mutations in the *pmr*(*pmr*A or *pmr*B) gene was 40.4% (23/57) in NTS isolates and 42% (22/50) in DEC isolates, resulting in alterations to amino acid sequences at various positions. Studies have shown that such mutations significantly contribute to AMR in bacteria, including *Salmonella*, which specifically confers resistance to polymyxin drugs, such as polymyxin B and E.^45^,^88^,^89^ These drugs are FDA approved for treating serious infections caused by multidrug-resistant gram-negative bacteria, especially those from the family Enterobacteriaceae.^90^ However, these antimicrobial agents are not commonly used in Ethiopia.

## Limitations of the Study

The present whole genome sequencing-based genotypic AMR analysis provides important information on the occurrence and spread of antimicrobial resistant strains of nontyphoidal *Salmonella* and diarrheagenic *E. coli* from various sources in Ethiopia. However, our study did not explore the mechanisms of AMR development in these bacteria to assess the impact of each point mutation identified at different nucleotide positions. This is because, in addition to acquired antimicrobial resistance genes (ARGs), some point mutations may occur naturally without adverse effects.

## Conclusion and Recommendations

Antimicrobial resistance poses a significant threat to public health globally, especially in low- and middle-income countries such as Ethiopia, where awareness towards AMR is limited. The current study identified acquired resistance genes and point mutations that mediate AMR to a wide range of drugs commonly used in Ethiopia, including aminoglycosides, tetracycline, folate pathway antagonists, and β-lactam agents. Interestingly, some ARGs were detected across all sample categories, with the most diverse ARGs encoding the β-lactam class. Moreover, some ARGs, such as *bla*_CTX-M-15,_ were found to confer resistance to up to nine antimicrobial agents, whereas *aac*(6′)-Iaa predicted that NTS isolates were completely resistant to tobramycin and amikacin. The findings of this study strongly suggest the presence of widespread MDR in both NTS and DEC strains in Ethiopia. Therefore, collaborative multisectoral measures, including enhancing and enforcing national and regional guidelines for prudent use of antimicrobials, are essential. Furthermore, robust, comprehensive, and continuous surveillance of AMR is needed to monitor the emergence and spread of drug-resistant pathogens in the country.

## Data Availability

The data used in this study are presented in the document, and supplementary files are also included. The raw sequences have been submitted to the European Nucleotide Archive (ENA) under accession number PRJEB73590.
